# Supplementary material for: Gut microbiota influences pathological angiogenesis in obesity‐driven choroidal neovascularization
Source: EMBO Mol Med. 2016 Nov 15;8(12):1366–79. doi: 10.15252/emmm.201606531 (PMC5167134; doi:10.15252/emmm.201606531)
Supplement: Supplementary file 1 — Expanded View Figures PDF [file EMMM-8-1366-s001.pdf]

Expanded View Figures

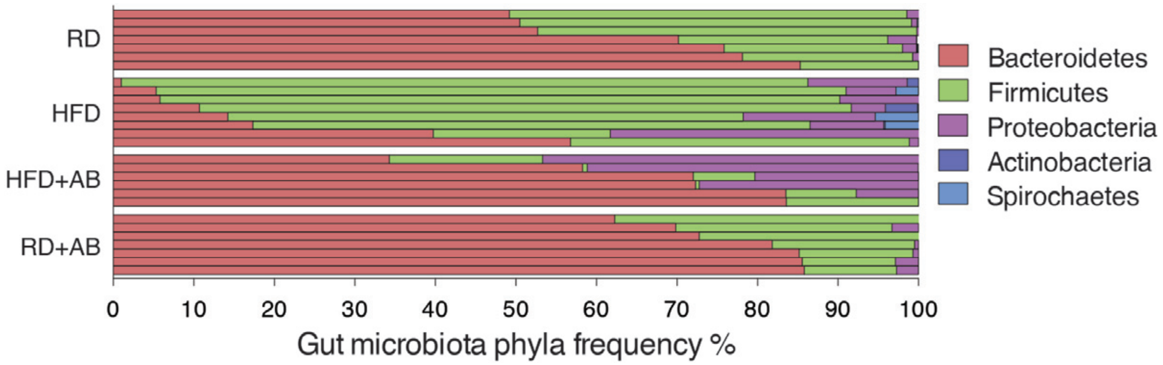

Figure EV1. Mice on HFD have shifted ratios of commensal gut microbes.
Relative abundance of bacterial phyla in gut microbiota of RD-fed mice with vehicle, HFD-fed mice with vehicle, HFD-fed mice with neomycin, and RD-fed mice with neomycin, shown per sample; *n* = 7 (RD), 8 (HFD), 6 (HFD+AB), 7 (RD+AB).

© 2016 The Authors

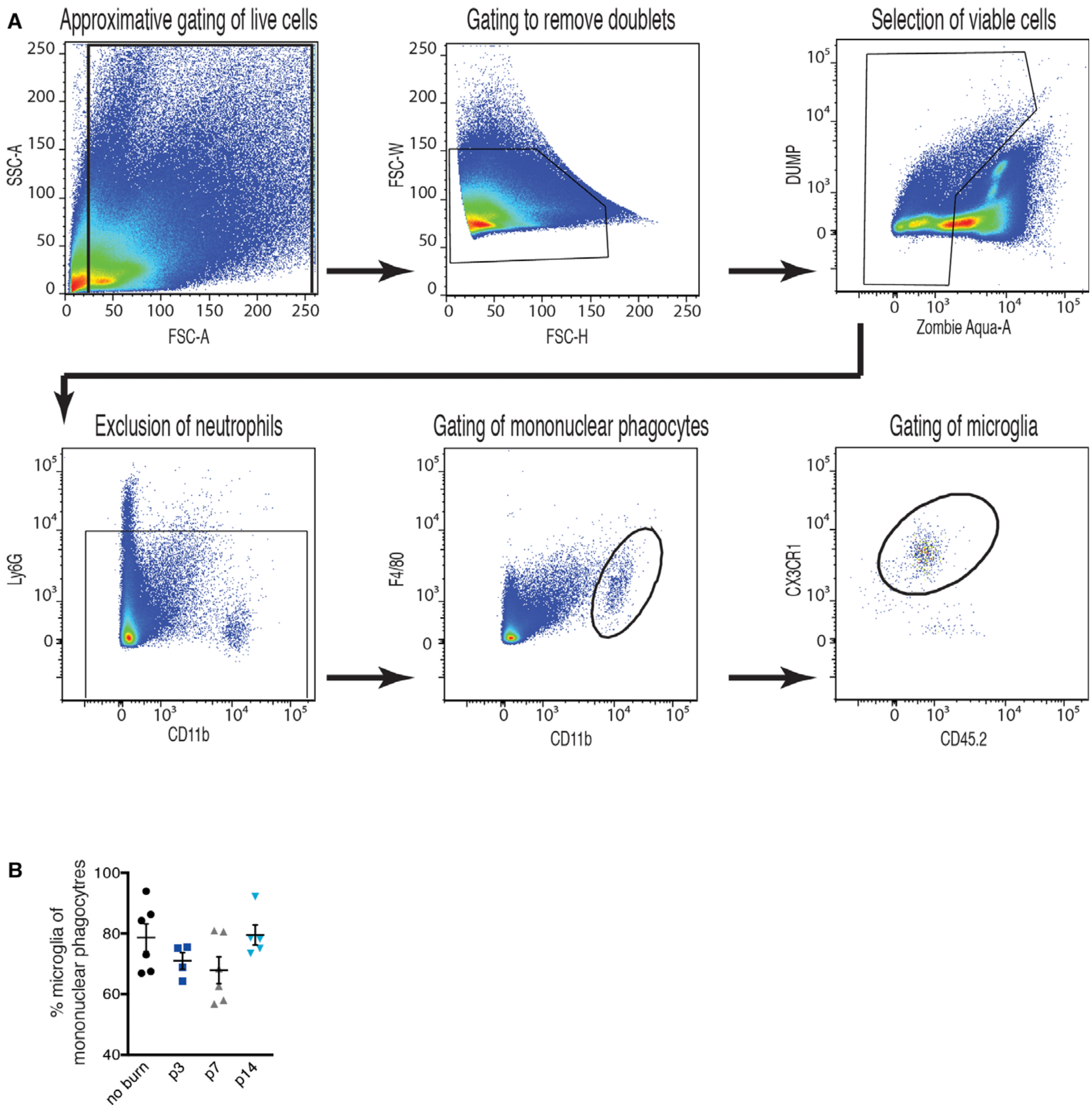

Figure EV2. FACS gating scheme and relative expression of MPs.

A Gating scheme explaining the identification of the Ly6G^{-}, F4/80^{+}, CD11b^{+} mononuclear phagocytes and the Ly6G^{-}, F4/80^{+}, CD11b^{+}, CX3CR1^{hi} CD45^{lo} microglia in retinas and sclera–choroid–RPE cell complexes. The gating scheme was as follows: gating of live cells, removal of doublets, selection of viable cells, exclusion of neutrophils, gating of mononuclear macrophages, and gating of microglia.

B Quantification of overall proportion of microglia within the MP population; n = 6 (no burn), 4 (p3), 6 (p7), 5 (p14). Error bars represent mean ± SEM. Each "n" represents one mouse.

 © 2016 The Authors

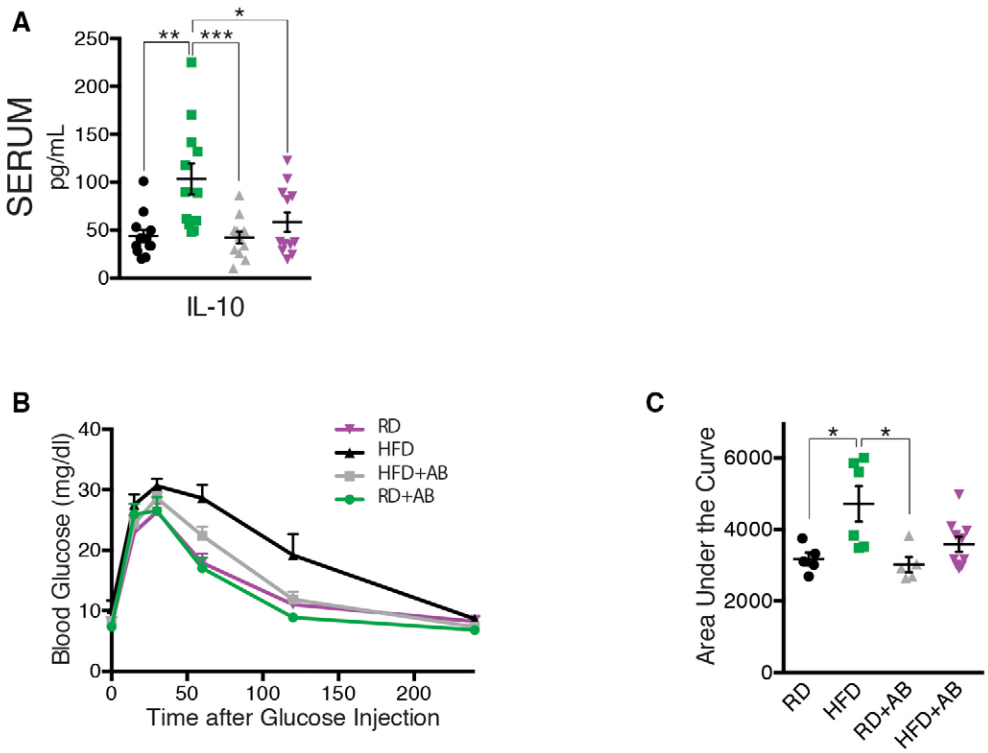

Figure EV3. Serum IL-10 is induced with HFD and neomycin treatment in HFD mice improves glucose tolerance.

A Serum IL-10 profile; n = 12 for all groups; CI 95%.

B Blood glucose (mg/dl) following intraperitoneal injection of glucose (2 mg/kg) in RD, HFD, HFD+AB, and RD+AB mice. n = 5 (RD), 6 (HFD), 5 (HFD+AB), 10 (RD+AB); CI 95%.

C Area under the curve (AUC) of the same groups as in (B).

Data information: All comparisons between groups are analyzed using one-way analysis of variance (ANOVA) and Tukey's multiple comparisons test; *$P < 0.05$, **$P < 0.01$, ***$P < 0.001$; error bars represent mean ± SEM. Each "n" represents one mouse; CI, confidence interval.

© 2016 The Authors

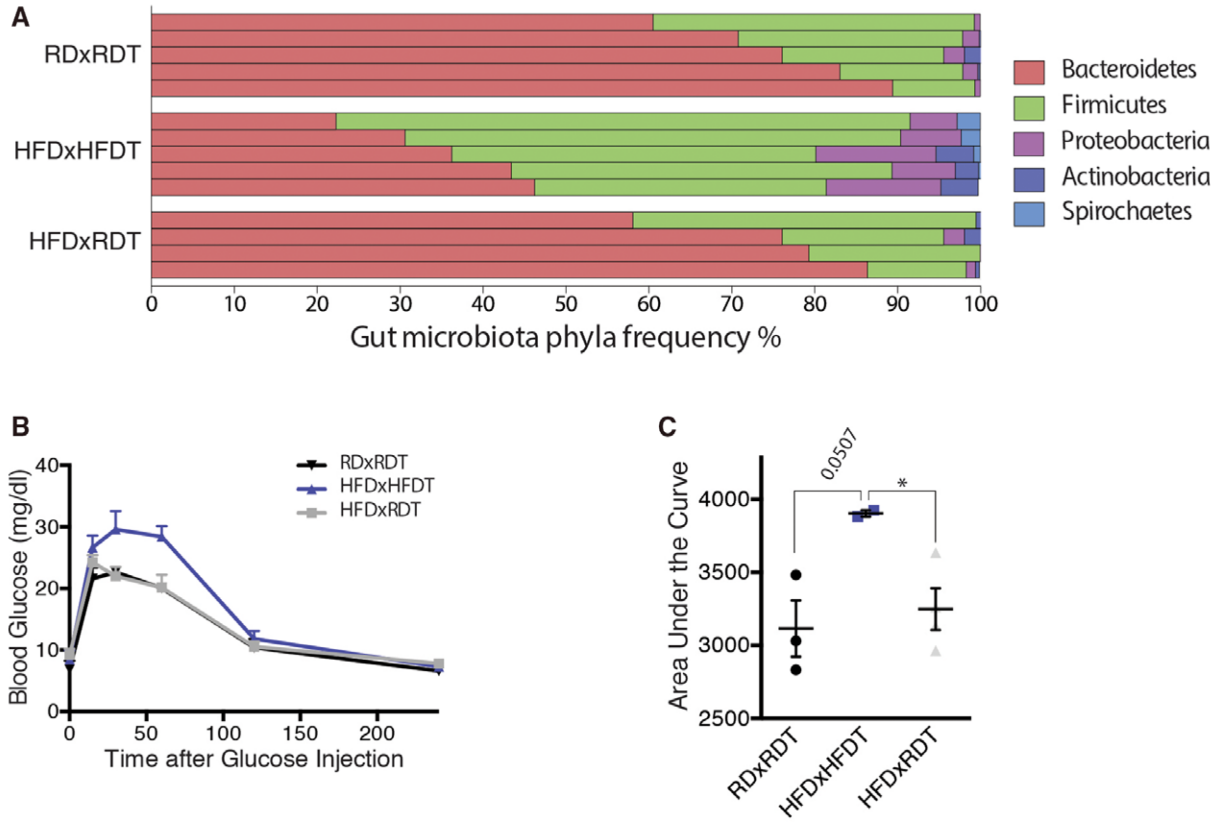

Figure EV4. Transplantation of RD feces into HFD mice rebalances gut microbiota and improves glucose tolerance.

A Relative abundance of bacterial phyla in gut microbiota of RDxRDT, HFDxHFDT, and HFDxRDT mice, shown per sample; n = 5 (RDxRDT), 5 (HFDxHFDT), 4 (HFDxRDT).

B Blood glucose (mg/dl) following intraperitoneal injection of glucose (2 mg/kg) in RDxRDT, HFDxHFDT, and HFDxRDT mice. n = 3 (RDxRDT), 2 (HFDxHFDT), 4 (HFDxRDT); CI 95%.

C Area under the curve (AUC) of the same groups as in (B). All comparisons between groups are analyzed using one-way analysis of variance (ANOVA) and Tukey's multiple comparisons test; *$P < 0.05$; error bars represent mean ± SEM. Each "n" represents one mouse; CI, confidence interval.

© 2016 The Authors
